# Unexpected Acceleration in Treprostinil Delivery Administered by a Lenus Pro® Implantable Pump in Two Patients Treated for Pulmonary Arterial Hypertension

**DOI:** 10.3389/fmed.2020.539707

**Published:** 2020-10-30

**Authors:** Garance Kopp, Anne-Lise Hachulla, Stéphane Noble, Aurélien Bringard, Paola M. Soccal, Maurice Beghetti, Frédéric Lador

**Affiliations:** ^1^Division of Pneumology, University Hospitals of Geneva, Genève, Switzerland; ^2^Division of Radiology, University Hospitals of Geneva, Genève, Switzerland; ^3^Pulmonary Hypertension Program, University Hospitals of Geneva, Genève, Switzerland; ^4^Division of Cardiology, University Hospitals of Geneva, Genève, Switzerland; ^5^Medical Faculty, University of Geneva, Genève, Switzerland; ^6^Division of Anesthesiology, Pharmacology, and Intensive Care, University Hospitals of Geneva, Genève, Switzerland; ^7^Pediatric Cardiology Unit, Children's Hospital, University Hospitals of Geneva, Genève, Switzerland

**Keywords:** pulmonary arterial hypertension, prostacyclin analogs, implantable pump, treprostinil delivery, internal device

## Abstract

Intravenous treprostinil administration by an implantable pump is an attractive option for pulmonary arterial hypertension (PAH) treatment and is the subject of recent publications. Short-term studies are promising, but there is still a lack of long-term prospective data. We analyzed the treprostinil flow rate administered by the Lenus Pro® implantable pump in 2 patients suffering from PAH during follow-up times of respectively 4.2 and 3 years. The flow rate delivered by the pumps in these 2 patients exceeded the manufacturer admitted margin of error within 2 years and continued to increase to reach, respectively, 158 and 120% of the expected flow rate at the end of the follow up. In one case, the implantable pump had to be removed for this reason. The *ex-vivo* flow rate of the withdrawn pump determined in the laboratory reached 173% of the predicted value. This correlated with the *in-vivo* measurement, which suggests a continuous flow increase even after pump removal and without treprostinil use. Spontaneous flow increase from such an implantable pump is a potentially major pitfall, which needs to be identified and actively managed by the responsible clinicians.

## Introduction

The prostacyclin pathway is a major target for treatment of pulmonary arterial hypertension (PAH), recommended for high-risk patients (1). Due to their short half-lives, prostacyclin analogs are usually administrated by continuous intravenous (IV), subcutaneous (SC) or intermittent inhaled routes. Treprostinil has a longer half-life and is much more stable at room temperature than epoprostenol permitting oral, SC or IV route administration. Its use has shown improvement in 6-min walk distance, functional class and pulmonary hemodynamics compared to placebo (2–4). The use of oral treprostinil is limited to first line therapy in non-high risk patients, due to its lesser efficacy than parenteral treprostinil (4). Unfortunately SC treprostinil administration is often limited by significant local side effects (2). Continuous IV administration is traditionally performed by central venous catheter connected to an external pump.

**Figure 1:**
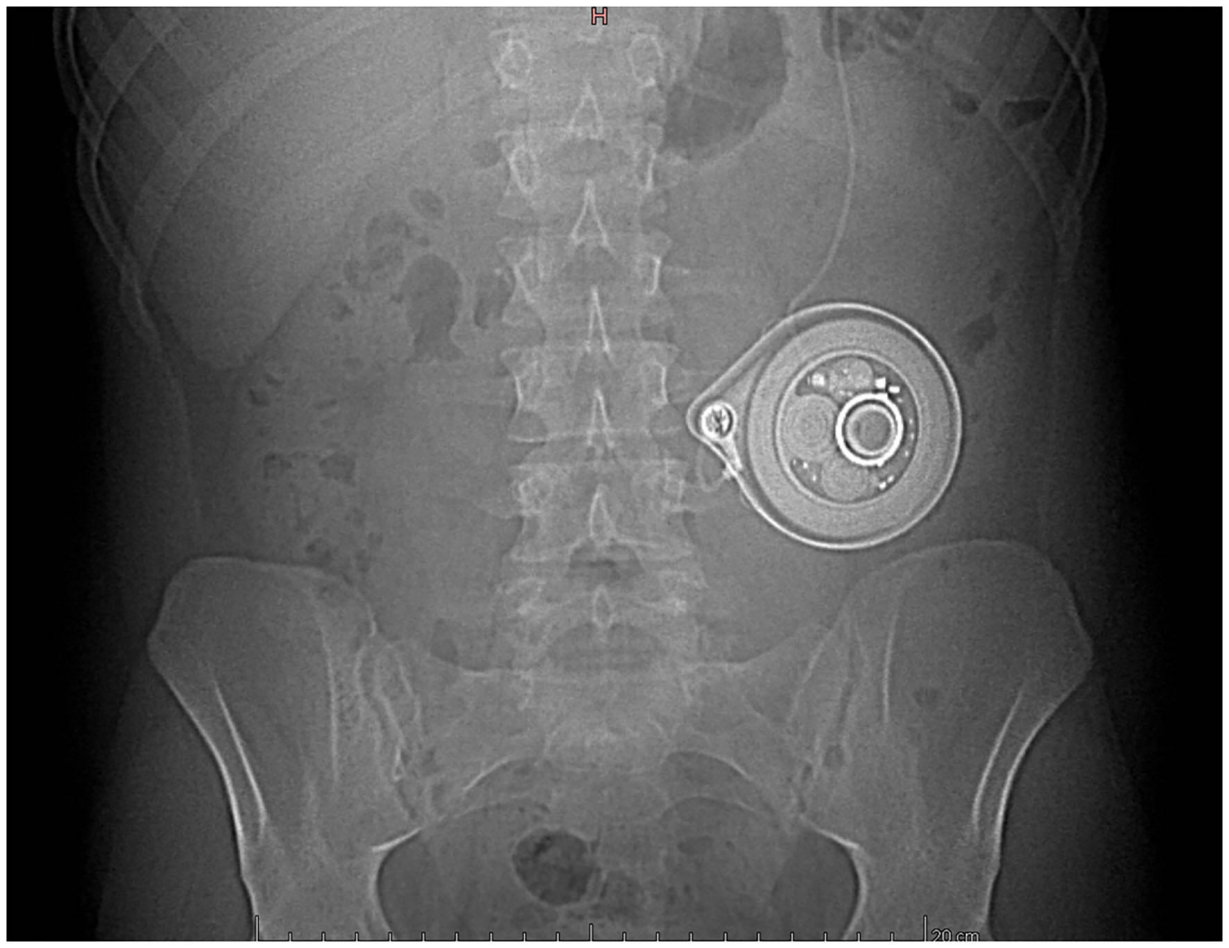
The pump (Lenus Pro®, 20 ml) implanted in the left hypochondria for intravenous treprostinil delivery (patient 1).

The development of an implantable pump for continuous IV treprostinil administration by Tricumed Medizintechnik GmbH (LenusPro® pump, approved in Europe in 2009) has attracted scientific attention as it may offer less restrictive use by patients as well as reducing catheter-related bloodstream infection rates (5–7). The Lenus Pro® pump contains a micro-infusion system that operates by pressurized gas storage in a titanium tank which is accessible by a puncturable silicone interface directly under the skin. Two pump models are available, one with a 20 ml capacity, the other with a 40 ml capacity. The device is usually implanted in the abdominal subcutaneous tissue and connected to a tunneled catheter accessing the superior vena cava (Figure 1). The flow is regulated by a glass capillary chip intended to guarantee a constant flow rate (1.3 ml/day), irrespectively of gas pressure. As the flow rate is fixed, drug titration is performed by adjusting treprostinil concentration. Percutaneous refills are usually performed every 14–28 days, depending on the size of the pump.

Two small prospective studies assessed periprocedural and 6-month safety, respectively, reporting good clinical results when using IV treprostinil administered by this pump (5, 6). We share here our experience with 2 cases receiving intravenous treprostinil via a LenusPro® implantable pump during a follow-up of, respectively, 4.2 and 3 years.

## Materials and Methods

The residual treprostinil volume in the pump was measured by a PAH specialized nurse before each refill procedure for the two patients treated by IV treprostinil administered by an implantable pump, as recommended by the manufacturer. The time between refill procedures and the volume administered were also reported in the registry by the nurse. Using these data we calculated the treprostinil flow rate between each refill procedure, for both patients during the follow-up period.

The *ex-vivo* flow rate of the withdrawn pump was determined in the laboratory by refilling it with saline solution (0.9 % NaCl) and keeping it in a constant 37°C water bath for 7 days. The flow rate was established by weighting (to 100 μg precision) at 24 h intervals a 5 ml polystyrene tube (352058 Becton-Dickinson) filled by the pump.

Written informed consent was obtained from the individuals for the publication of any potentially identifiable images or data included in this article.

## Cases

**Figure 2:**
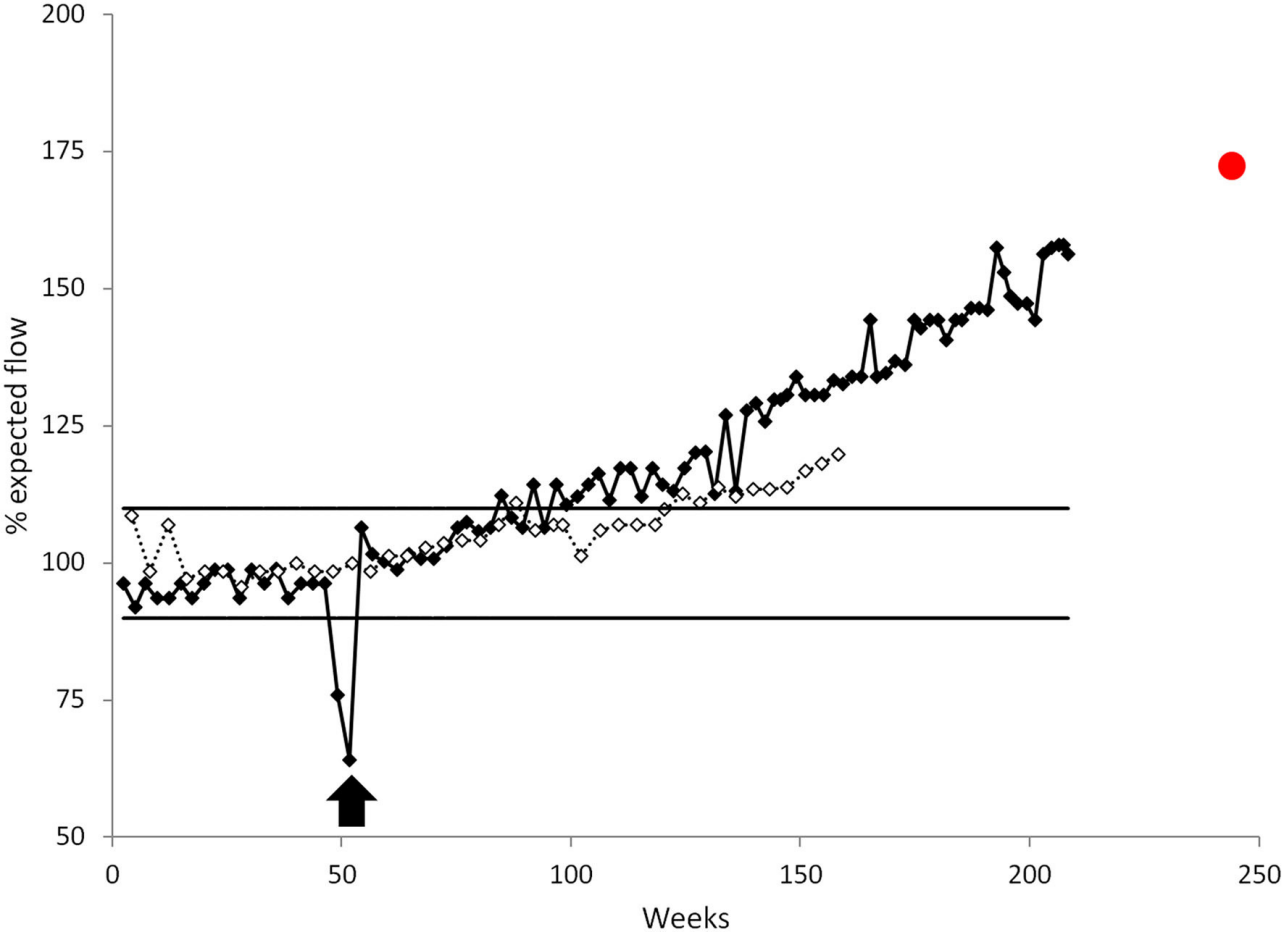
Treprostinil delivery flow rate by the LenusPro® pump for patient 1 (black) and patient 2 (white) expressed relative to the expected calibrated flow. For patient 1, a catheter thrombosis resulted in a dramatic flow decrease that was treated by a standard repermeabilization procedure (black arrow). Horizontal lines represent the ±10% accepted flow error. The red dot represents the *ex-vivo* flow rate of the withdrawn pump determined in experimental condition by refilling it with a 20 ml saline solution.

The first case is a 47-year-old female patient suffering from drug-induced PAH. Under combined dual therapy (sildenafil, bosentan) the initial evolution was favorable. Due to clinical and hemodynamic deterioration, treatment of SC treprostinil was introduced later. The tolerance of the latter quickly became limited by pain at the injection site, justifying a switch to IV treatment. The patient being reluctant to IV access with external pump an implantable pump was proposed (LenusPro® 20 ml, Figure 1). The target dosage was reached in 2 months (47 ng/kg/min) while the clinical improvement allowed stabilization in functional class II. One year after implantation a central catheter thrombosis occurred resulting in a 40% flow rate decrease (see Figure 2). This was only discovered by the observation of an increased residual volume in the pump whereas the electronic flow rate sensor should have triggered an alarm in such a case. The catheter thrombosis was successfully treated by gentle rinsing with a heparin solution under fluoroscopic control by contrast injection, according to the manufacturer's guide. During the 4 years of follow-up we observed a gradual decrease in the residual treprostinil volume at refilling resulting in a shorter period between refill procedures.

The pump is calibrated by the manufacturer to a constant flow rate (1.04 ml/day ± 10%) whereas 4.2 years after implantation the observed flow rate had increased to 1.64 ml/day (77.4 ng/kg/min) which is a 58% flow increase above the predicted value (Figure 2). The increase in the treprostinil delivery flow rate resulted a proportional drug-dose increase that was, fortunately, well tolerated but induced a significant cost increase. The pump was removed and replaced by a 40 ml model due to the patient still being reluctant to other modalities of treatment and still being stabilized on this therapy. The *ex-vivo* flow rate of the withdrawn pump was established at 1.797 ± 0.017 ml/day, corresponding to a 73% flow increase over the expected value (Figure 2). This suggests a continuous flow increase even after pump removal and independent of treprostinil use. Considering this particular case, it was considered that the dose increase may have contributed to the progressive need of the PAH evolution and that it may have participated to maintain the patient in a stable condition.

The second case is a 22-year-old male patient followed since childhood for idiopathic PAH. The clinical course was initially favorable under inhaled iloprost monotherapy sequentially combined with bosentan and, later, sildenafil due to hemodynamic deterioration. Despite the triple therapy the clinical and hemodynamic response was unsatisfactory. The iloprost treatment was successfully changed from inhaled to IV route delivered by a central venous catheter. Following that time the patient developed numerous catheter-related infections requiring hospitalizations and central line replacements. In order to reduce the risk of infection an implantable pump (LenusPro® 40 ml) was fitted.

As with the first case we observed a progressive increase in treprostinil flow reaching 1.52 ml/day which is a 20% increase over the calibrated value of 1.27 ml/day (± 10%) (Figure 2). The drug dilution was adjusted at each refill procedure in order to counterbalance the increased flow rate and maintain a stability of treatment at 35–41 ng/kg/min without any adverse event or side effect related to the treatment. Despite these measures, further hemodynamic evaluation concluded that the PAH was worsening under maximal treatment. The patient was referred for a lung transplant but died suddenly whilst still on the waiting list.

## Discussion

The currently available studies of the Lenus Pro® pump have drawn justifiable attention with good results reported and without significant short-term complications or safety issues (5, 6). Nonetheless the studies included small collectives and only two of them were prospective.

Interestingly a recent retrospective study including 129 patients with pulmonary hypertension (PH) treated by IV treprostinil administered by the Lenus Pro pump® has shown 82 complications after a median follow-up of 19 months. Most of these events were non-infectious catheter-related complications. As in the cases previously described, flow rate increases occurred in five patients. Four of them required pump replacement for this reason. Following treprostinil flow rate increase, two patients developed acute cardiac decompensation requiring intensive care unit admission. One patient underwent pump replacement. In the other patient, the treprostinil dose was adjusted to the higher flow rate without explantation (7). An unexpected increase in the flow rate administered by a LenusPro pump was also reported in a 14 years old patient (8).

In their multicentric study, Ewert et al. reported a septum defect of a Lenus Pro pump in one patient (6). This resulted in a severe overdose with subsequent hypotension, necessitating pump replacement. This septum defect was considered by the authors likely to be due to improper use of non-approved needles for refilling the pump.

This highlights the importance of maintaining a constant prostacyclin analog flow rate for patient stability and improvement as previously described in another study (9).

A warning has been published in 2013 by the manufacturer reporting occurrences of increased flow of the LenusPro® pump after several years of use. It is speculated that the molecule or its solvent provokes an increase in the cross-section of the glass capillary canal of the chip and therefore an augmentation of flow by 10%, especially when pumps are used for longer periods of time (2–4 years). The corrective action was introduced by the new development of a chip canal with a more resistant glass (10).

Nevertheless, an unexpected increase in the drug administration rate was reported with a Lenus Pro implantable pump coming from upgraded series, i.e., after the 2013 safety note of the manufacturer (11). This increase from 1.3 to 1.7 ml/day resulted in symptoms of deterioration of exercise capacity and fatigue without hemodynamic collapse. The second pump of the first described patient of the present work was also introduced after 2013, demonstrating that the corrective action introduced may not have been sufficient.

Recently, the variance of the fixed flow rate during long-term follow-up has been addressed on 126 patients (12) during a median follow-up of 12 months. Based on 2,853 refills, the relative flow rate deviation between each individual refill was between −10% and +10% in 94.5% of cases. However, three refill cases (0.1%) had a relative flow rate deviation of more than 40% from the previous refill (one case of −40% and two cases of +40%).

SynchroMed® II (Medtronic) is the second implantable pump currently approved for IV treprostinil administration in patients with PH. Unlike the Lenus Pro® pump, SynchroMed® II pump is battery-driven and has an adjustable flow rate, between 0.048 and 24 ml/day. Nonetheless, recent studies reported decreased flow rate and flow rate accuracy over time with this device (13, 14). Gomberg-Maitland et al. reported also eight pump failures events (13). One of them was undetected, leading to subsequent death of the patient. After this, pump design have been implemented, addressing many of the observed pump failures and motor stalls. Later, a programmable battery-driven model (Siromedes® pump) with adjustable flow has been developed by the German company Tricumed and approved in 2014. The three devices have similar use and implantation procedures.

The use of an implantable pump for IV treprostinil administration in PAH management is attractive, but the unexpected increase in the rate of treprostinil delivery by the Lenus Pro® pump is a major issue.

Such an increase is potentially life threatening and must be detected and actively managed by the responsible clinicians. This therapy should be administered in PH specialized centers by clinicians familiarized with the device. Reporting the time between refill procedures, the residual volume and the volume administered at each refill procedure should allow early detection of any derivation from the target flow rate and anticipate potential pump failure.

As an alternative option of removing and replacing the defective pump, adjustment of the drug dilution has to be considered, as long as the flow deviation from the factory setting can be anticipated.

This report is limited to the description of only two cases. Nevertheless, the frequency of adverse events such as previously described in our cases could have been underestimated in the studies actually available, due to the retrospective bias. Prospective studies are still needed to assess the long-term safety and efficacy of treprostinil administration by this device.

## Data Availability Statement

The datasets generated for this study are available on request to the corresponding author.

## Ethics Statement

We hereby confirm that written informed consent was obtained from the individuals for the publication of any potentially identifiable images or data included in this article.

## Author Contributions

GK, MB, and FL contributed conception and design of the article. AB and FL performed the analysis of the withdrawn pump. GK wrote the first draft of the manuscript. MB, AB, and FL wrote sections of the manuscript. All authors contributed to the article and approved the submitted version.

## Conflict of Interest

MB has served as consultant and/or advisory board member for Actelion, Bayer-Schering, Lilly, GlaxoSmithKline, Novartis, MSD, and Pfizer Inc. and has received investigator-initiated research funding from Actelion and Bayer-Schering. FL has served as consultant and/or advisory board member for Novartis, MSD, and Bayer-Schering, has received conference fees from Actelion, Bayer-Schering, Lilly, GlaxoSmithKline, Novartis, and Pfizer Inc., and has received an educational grant from GlaxoSmithKline. The remaining authors declare that the research was conducted in the absence of any commercial or financial relationships that could be construed as a potential conflict of interest.
